# Role of Tumor Molecular Profiling With FoundationOne®CDx in Advanced Solid Tumors: A Single-Centre Experience From Romania

**DOI:** 10.7759/cureus.50709

**Published:** 2023-12-18

**Authors:** Ana Maria Popa, Mihaela Andreea Stejeroiu, Cristian Iaciu, Mihaela Olaru, Cristina Orlov Slavu, Andreea Parosanu, Ioana Miruna Stanciu, Cristina Pirlog, Simina Pavel, Cornelia Nitipir

**Affiliations:** 1 Medical Oncology, Elias Emergency University Hospital, Bucharest, ROU; 2 Oncology, Carol Davila University of Medicine and Pharmacy, Bucharest, ROU

**Keywords:** cancer genomic profiling, next generation sequencing (ngs), oncology, personalized treatment, targeted therapy

## Abstract

Background

In the field of precision oncology, comprehensive genomic profiling tests play a very important role by providing a complex understanding of the molecular characteristics of malignant tumors. Therefore, next-generation sequencing (NGS) has become a valuable tool in various aspects of cancer care from diagnosis and monitoring to treatment selection and personalized cancer treatment. Our aim was to evaluate the role of tumor molecular profiling in tailored treatment selection.

Methods

In our study, we conducted a retrospective analysis to assess the practicality of utilizing NGS testing in patients with metastatic solid tumors. The genomic testing was performed on blood or tissue samples from a fresh biopsy, less than six months old, and the expression of programmed death-ligand 1 was evaluated by immunohistochemistry.

Results

A total of 75 tests were performed on 66 patients between 2019 and 2022, with a success rate of 80%. The most common pathologies were gastro-intestinal tract cancer (26%), breast cancer (14%), non-small cell lung cancer (11%), and pancreatic cancer (11%). There were 9% liquid biopsies and 91% tissue biopsies. From all 66 patients tested, 55 had at least one genetic alteration. The most frequent genetic alteration found was TP53 (n=32) followed by KRAS (n=15) and BRCA1/2 (n=12) mutations. There were nine patients tested (14%) that presented a high tumor mutational burden, and only one patient presented high microsatellite instability. There were 37 patients (56%) with actionable alterations found from which 14 received matched therapy and four patients were enrolled in clinical trials. The NGS testing played a significant role in determining the next therapeutic strategy in 20 out of 66 patients (30.3%).

Conclusion

From all the patients included in our analysis, 83% had at least one mutation that is known to be of pathogenic significance but only 23% received treatment selected by the analysis of the tumor’s genome, and only 6% were included in a clinical trial. This moderate success of personalized medicine using NGS testing highlights the importance of evaluating the factors that could lead to further improvement.

## Introduction

The idea of DNA sequencing of the cancer cells’ genome for detecting the driver mutations and for a better understanding of individual cancer care emerged with the beginning of the significant advancements in genomic research. This concept was reinforced by many studies such as those conducted by Stratton et al. and Vogelstein et al., which shed light on the critical role of genetic mutations in cancer [1,2]. The concept of identifying the key mutations and molecular drivers of the disease became the foundation for the application of DNA sequencing technologies in oncology. By understanding the genomic landscape of tumors, the clinicians are able to apply tailored strategies and offer personalized therapies to cancer patients.

In recent years, there have been significant advancements in molecular research regarding cancer care. Among the breakthroughs, next-generation sequencing (NGS) has surfaced as a new tool for diagnosis, treatment and management of cancer patients. As these new technologies continue to evolve and become more accessible, it is important to understand their actual role in cancer care and to use them properly for obtaining the best outcomes for oncologic patients.

Some recent studies focusing on patients with refractory oncologic disease demonstrated promising outcomes using DNA sequencing for the identification of targetable genomic alterations. This approach enabled the administration of personalized treatments to eligible patients, but there is still the need for further research to clearly determine some key aspects in the field of genomic profiling. These include identifying the appropriate candidates, the optimal time to perform genomic profiling throughout the disease progression, exploring the most reliable technique and also strategies to make tumor profiling more cost-effective and accessible [3-5].

As there are advancements in the field of molecular oncology, and the genomic sequencing technologies become more and more performant and accessible, new challenges for clinicians arise. It is now a challenge for the medical oncologists to determine the relevance, in the clinical practice, for every particular result of the multigene panel sequencing test. The European Society for Medical Oncology (ESMO) developed a classification system called ESMO Scale for Clinical Actionability of Molecular Targets (ESCAT). This scale has the purpose of helping medical professionals to choose from the potential targets, the ones with evidenced clinical value. This classification system is updating consistently as new evidence is produced by clinical studies. ESCAT became a global classification system, offering a common language for all the oncology professionals [6].

The absence of specific national guidelines poses a challenge for clinicians in effectively utilizing the emerging genomic profiling technology to optimize outcomes for cancer patients. The lack of such guidance has prompted us to share our preliminary experience of genomic profiling in a cohort of 66 cancer patients who had undergone prior treatment. The purpose of this retrospective study was to investigate the feasibility and utility of clinical application of genomic testing in metastatic pre-treated patients and to identify potential targetable alterations.

## Materials and methods

Patients

We conducted an observational retrospective, monocentric study that included 66 patients who underwent genetic testing between 2019 and 2022. The inclusion criteria for the patients treated in our clinic were as follows: age over 18 years, histopathologically confirmed solid tumor malignancy, metastatic disease, at least one line of treatment, a fresh biopsy (less than six months old) from a progressive lesion and an Eastern Cooperative Oncology Group (ECOG) performance status (PS) between 0 and 2. All of them must have agreed to perform the NGS testing. The exclusion criteria were as follows: age under 18 years, absence of histopathological confirmation of the disease, incomplete medical history or clinical data and an ECOG performance status of 3 or 4 at the moment of the confirmation of the metastatic disease. Almost all of them progressed after two or more lines of therapy and had few therapeutic options left.

Ethical aspects

The current investigation adhered to both national and international research ethics guidelines and received approval from the Ethics Committee of the Elias University Emergency Hospital in Bucharest, Romania (approval no. 123112023-1). All the authors signed a data confidentiality agreement and consent to use the data for scientific purposes, and all patients signed an informed consent that enabled us to use their data for scientific purposes. Throughout the project's duration, the investigators adhered to ethical and medical deontology guidelines, encompassing both institutional standards and national regulations.

Molecular profiling and immunohistochemical staining

The genomic testing was performed on blood or tissue samples from a fresh biopsy, less than six months old, and the expression of programmed death ligand 1 (PD-L1) was evaluated by immunohistochemistry.

The genomic profiling test used for analysis was performed through FoundationOne®CDx panel (Foundation Medicine, Cambridge, MA) that analyses the DNA of cancer cells. Genomic testing may identify both germline and somatic mutations, but does not differentiate between the two of them. The test evaluates four major categories of genomic alterations (base substitutions, insertions and deletions, copy number alterations and rearrangements) in 324 well-established genes associated with cancer. Additionally, it assesses tumor mutational burden (TMB) and microsatellite instability (MSI), providing valuable information for determining eligibility for immunotherapy. The test also reports high levels of loss of heterozygosity (LoH), which may indicate homologous recombination deficiency (HRD+), guiding the potential use of poly-ADP ribose polymerase (PARP) inhibitors [7].

The gene alterations detected through the FoundationOne®CDx panel are classified into two categories: known or likely pathogenic variants documented in the Foundation Medicine database, which encompasses entries from the Catalogue of Somatic Mutations in Cancer (COSMIC) database, and variants of unknown significance (VUS), all of which are discussed in detail in the FoundationOne®CDx report [7]. The genes tested by FoundationOne®CDx are detailed in Table 1.

**Table 1:** Genes with full coding exonic regions included in FoundationOne®CDx for the detection of substitutions, insertions and deletions (indels), and copy number alterations *Genes with copy number alteration reporting are limited to CDx variants when using the CoExtraction method Source: Table from www.foundationmedicine.com (accessed on November 12, 2023)

Genes										
ABL1	BRAF	CDKN1A	EPHA3	FGFR4	IKZF1	MCL1	NKX2-1	PMS2	RNF43	TET2
ACVR1B	BRCA1*	CDKN1B	EPHB1	FH	INPP4B	MDM2	NOTCH1	POLD1	ROS1	TGFBR2
AKT1	BRCA2*	CDKN2A	EPHB4	FLCN	IRF2	MDM4	NOTCH2	POLE	RPTOR	TIPARP
AKT2	BRD4	CDKN2B	ERBB2*	FLT1	IRF4	MED12	NOTCH3	PPARG	SDHA	TNFAIP3
AKT3	BRIP1*	CDKN2C	ERBB3	FLT3	IRS2	MEF2B	NPM1	PPP2R1A	SDHB	TNFRSF14
ALK	BTG1	CEBPA	ERBB4	FOXL2	JAK1	MEN1	NRAS	PPP2R2A	SDHC	TP53
ALOX12B	BTG2	CHEK1*	ERCC4	FUBP1	JAK2	MERTK	NT5C2	PRDM1	SDHD	TSC1
AMER1	BTK	CHEK2*	ERG	GABRA6	JAK3	MET	NTRK1	PRKAR1A	SETD2	TSC2
APC	C11orf30	CIC	ERRFI1	GATA3	JUN	MITF	NTRK2	PRKCI	SF3B1	TYRO3
AR	CALR	CREBBP	ESR1	GATA4	KDM5A	MKNK1	NTRK3	PTCH1	SGK1	U2AF1
ARAF	CARD11	CRKL	EZH2	GATA6	KDM5C	MLH1	P2RY8	PTEN*	SMAD2	VEGFA
ARFRP1	CASP8	CSF1R	FAM46C	GID4 (C17orf39)	KDM6A	MPL	PALB2*	PTPN11	SMAD4	VHL
ARID1A	CBFB	CSF3R	FANCA	GNA11	KDR	MRE11A	PARK2	PTPRO	SMARCA4	WHSC1
ASXL1	CBL	CTCF	FANCC	GNA13	KEAP1	MSH2	PARP1	QKI	SMARCB1	WHSC1L1
ATM*	CCND1	CTNNA1	FANCG	GNAQ	KEL	MSH3	PARP2	RAC1	SMO	WT1
ATR	CCND2	CTNNB1	FANCL*	GNAS	KIT	MSH6	PARP3	RAD21	SNCAIP	XPO1
ATRX	CCND3	CUL3	FAS	GRM3	KLHL6	MST1R	PAX5	RAD51	SOCS1	XRCC2
AURKA	CCNE1	CUL4A	FBXW7	GSK3B	KMT2A (MLL)	MTAP	PBRM1	RAD51B*	SOX2	ZNF217
AURKB	CD22	CXCR4	FGF10	H3F3A	KMT2D (MLL2)	MTOR	PDCD1	RAD51C*	SOX9	ZNF703
AXIN1	CD274	CYP17A1	FGF12	HDAC1	KRAS	MUTYH	PDCD1LG2	RAD51D*	SPEN	
AXL	CD70	DAXX	FGF14	HGF	LTK	MYC	PDGFRA	RAD52	SPOP	
BAP1	CD79A	DDR1	FGF19	HNF1A	LYN	MYCL	PDGFRB	RAD54L*	SRC	
BARD1*	CD79B	DDR2	FGF23	HRAS	MAF	MYCN	PDK1	RAF1	STAG2	
BCL2	CDC73	DIS3	FGF3	HSD3B1	MAP2K1	MYD88	PIK3C2B	RARA	STAT3	
BCL2L1	CDH1	DNMT3A	FGF4	ID3	MAP2K2	NBN	PIK3C2G	RB1	STK11	
BCL2L2	CDK12*	DOT1L	FGF6	IDH1	MAP2K4	NF1	PIK3CA	RBM10	SUFU	
BCL6	CDK4	EED	FGFR1	IDH2	MAP3K1	NF2	PIK3CB	REL	SYK	
BCOR	CDK6	EGFR	FGFR2	IGF1R	MAP3K13	NFE2L2	PIK3R1	RET	TBX3	
BCORL1	CDK8	EP300	FGFR3	IKBKE	MAPK1	NFKBIA	PIM1	RICTOR	TEK	

We analyzed PD-L1 expression by immunohistochemistry analysis (Ventana SP263; Ventana Medical Systems, Inc., Tucson, AZ). The result criteria were grouped into high positive (≥50% proportion of positive staining of at least 1+ intensity), moderate positive (25%-49% proportion of positive staining of at least 1+ intensity), low positive (1%-24% proportion of positive staining of at least 1+ intensity), negative (<1% proportion of positive staining of at least 1+ intensity, or no staining) and indeterminate (test reliability compromised). This information is summarised in Table 2.

**Table 2:** Result criteria of the PD-L1 immunohistochemistry analysis (Ventana SP263) PD-L1: programmed death ligand 1

Percentage of positive staining/intensity	Expression
≥50% proportion of positive staining of at least 1+ intensity	High positive
25%-49% proportion of positive staining of at least 1+ intensity	Moderate positive
1%-24% proportion of positive staining of at least 1+ intensity	Low positive
<1% proportion of positive staining of at least 1+ intensity, or no staining	Negative
Test reliability compromised	Indeterminate

The outcomes of the genomic profiling tests were reviewed and discussed during the multidisciplinary tumor board meetings. While our hospital does not have a dedicated molecular tumor board, we sought guidance from FoundationOne experts through a specialized platform that provides assistance in the treatment decision-making process, particularly for specific cases.

Statistical analysis

The data was processed using SPSS Statistics V.26 (IBM Corp., Armonk, NY). Progression-free survival (PFS), defined as the duration (in months) from the initial administration to radiological progression based on the Response Evaluation Criteria in Solid Tumours (RECIST), version 1.1 (RECIST 1.1), was assessed using the Kaplan-Meier method [8].

## Results

Between 2019 and 2022, we performed 75 FoundationOne tests on 66 patients. The turnaround time, which refers to the duration between obtaining informed consent and the physician receiving the test results, amounted to 21 days. The success rates of the tests, the patients characteristics and the characteristics concerning the tissue samples are listed in Tables 3-5. Out of the 75 tests performed, 60 tests were successful and 20% of the tests failed. The study cohort, comprising 66 patients, exhibited a diverse demographic and oncological profile. The median age of the patients was 57 years. Among them, 44% were male and 56% were female. The performance status varied, with 9% of patients presenting with an ECOG performance status of 0, 67% as ECOG PS 1, and 24% as ECOG PS 2. The cohort encompassed individuals with a range of cancer types, with notable proportions represented by gastrointestinal cancers (26%), breast cancer (14%), non-small cell lung cancer (11%) and pancreatic cancer (11%). Regarding the treatment received before the genetic testing, there was also a wide distribution as 18% of patients received one line, 37% received two lines, 29% received three lines, and 15% received four lines of treatment. Biopsy methods employed in the study included liquid biopsy for 9% of patients and tissue biopsy for the remaining 91%.

**Table 3:** Success rate of FoundationOne tests

No. of samples tested	No. of failed tests (%)	No. of tests successfully performed (%)
75	15 (20%)	60 (80%)

**Table 4:** Patient characteristics ECOG: Eastern Cooperative Oncology Group; PS: performance status; NSCLC: non-small cell lung cancer

Characteristic	Subset	No. of patients (%)
Sex	Male	29 (44%)
	Female	37 (56%)
ECOG PS	0	6 (9%)
	1	44 (67%)
	2	16 (24%)
Tumor site/type of cancer	Gastro-intestinal cancer	17 (26%)
	Breast cancer	9 (14%)
	NSCLC	7 (11%)
	Pancreatic cancer	7 (11%)
	Melanoma	5 (8%)
	Biliary tract cancer	4 (6%)
	Ovarian cancer	3 (4%)
	Urothelial cancer	3 (4%)
	Sarcoma	3 (4%)
	Other	8 (12%)
Lines of treatment	1	12 (18%)
	2	25 (37%)
	3	19 (29%)
	4	10 (15%)
Biopsy	Liquid	7 (9%)
	Tissue	68 (91%)

**Table 5:** Tissue sample characteristics

Characteristic	Subset	No. of patients (%)
Tissue source	Metastatic site	45 (61%)
	Primary tumor	29 (39%)
Metastatic site specimen	Hepatic	18 (40%)
	Lung	5 (11%)
	Peritoneum	5 (11%)
	Skin	4 (9%)
	Lymph nodes	4 (9%)
	Bone	3 (7%)
	Other	6 (13%)

The majority of specimens were derived from metastatic sites, comprising 61% of the total. Within the subgroup of metastatic site specimens, the distribution across various locations is as follows: 40% from hepatic metastases, 11% from lung metastases, 11% from the peritoneum, 9% from skin metastases, 9% from lymph nodes, and 7% from bone metastases. An additional 13% were sourced from other, less specified locations. Conversely, 39% of the specimens originated from primary tumors.

The results of tumor profiling regarding TMB and PD-L1 expression are described in Table 6.

**Table 6:** Tumor mutational burden and PD-L1 expression PD-L1: programmed death ligand 1

Characteristic	Number (%)
PD-L1 expression	
Not determined	11 (17%)
Negative	36 (54%)
Low positive	15 (23%)
Moderate positive	2 (3%)
High positive	2 (3%)
Tumor mutational burden	
Not determined	14 (21%)
High	9 (14%)
Low	43 (65%)

Regarding PD-L1 expression, a proportion of 17% of cases had undetermined levels. The majority, comprising 54%, exhibited a negative PD-L1 expression. Further stratification of positive cases revealed 23% with low positive expression, while a smaller percentage demonstrated moderate (3%) and high (3%) positive expression. In terms of TMB, 21% of cases had undetermined levels. Among those with determined TMB, a distinct pattern emerged, with 14% classified as having a high TMB and the majority, accounting for 65%, characterized by a low TMB.

**Figure 1:**
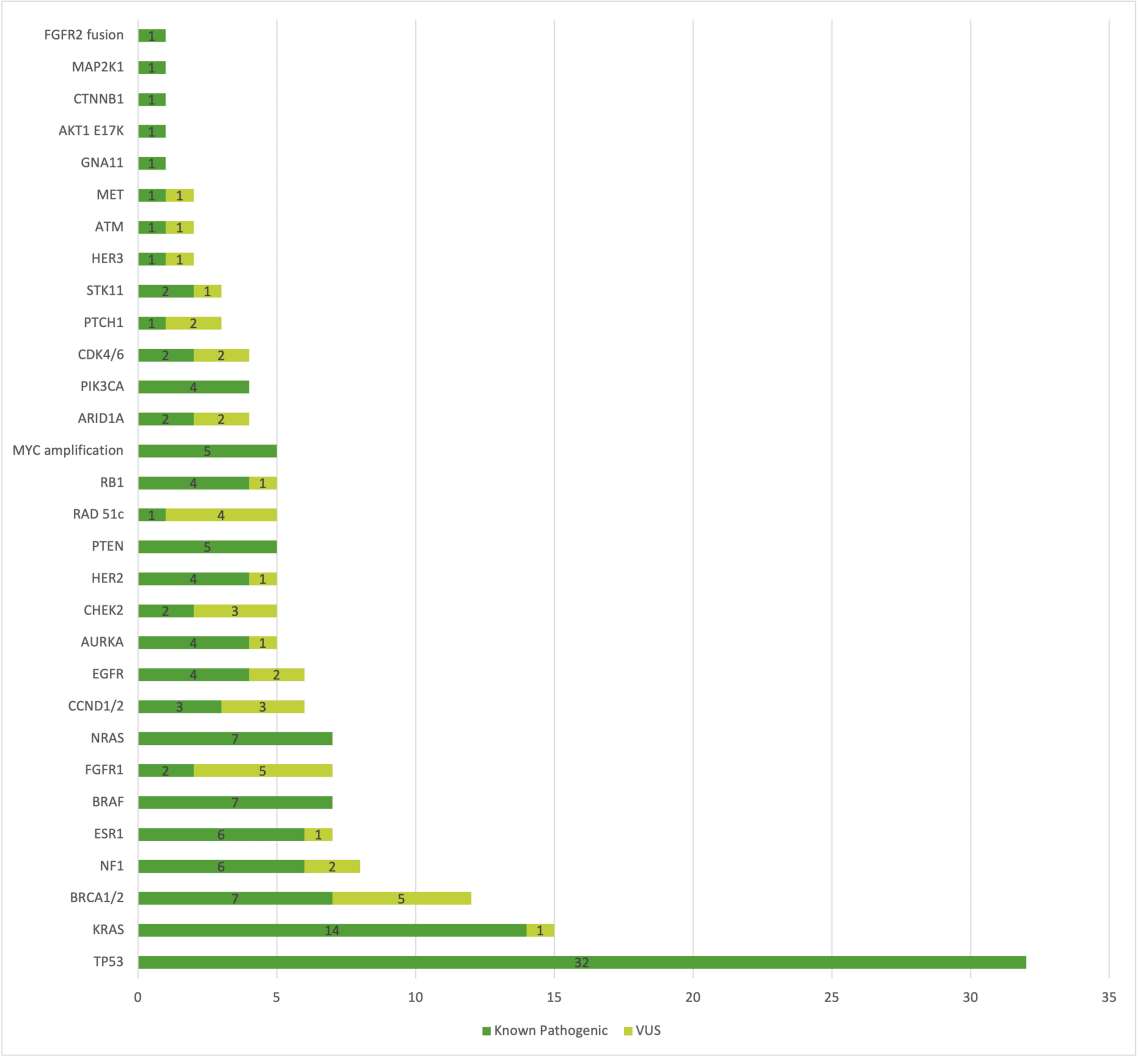
Distribution of the most frequent gene mutations (both known pathogenic and VUS) found in all the patients tested VUS: variants of unknown significance

The frequency of the genetic alterations found in our cohort is presented in Figure 1, with both the known pathogenic alterations of the genes and also the frequency of the mutations classified as variants of unknown significance. The most frequent genetic mutations were TP53 alterations.

**Figure 2:**
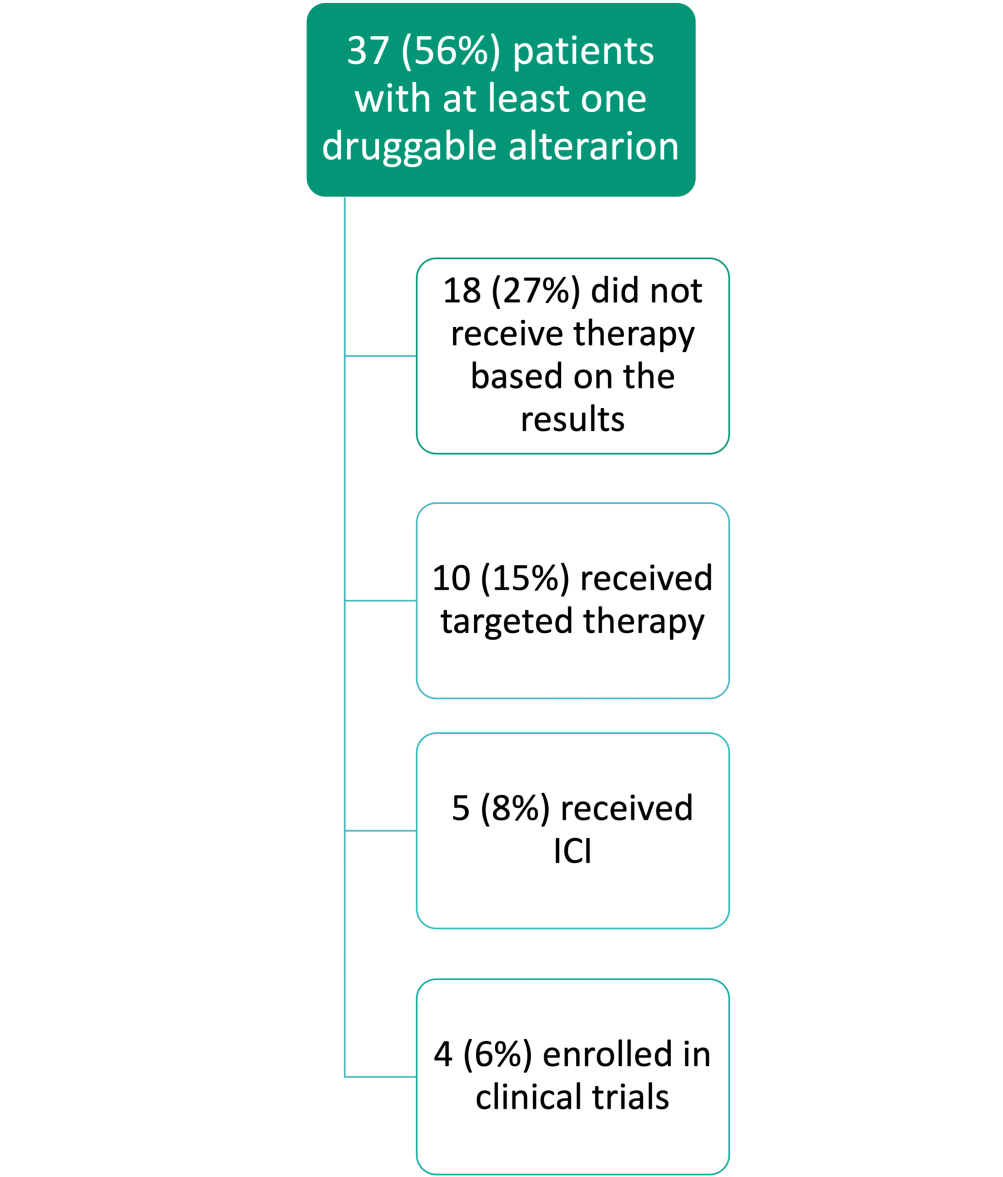
Courses of action for patients with actionable alterations ICI: immune checkpoint inhibitor

The data regarding patients who had druggable alterations and the recommended courses of action are presented in Figure 2. In 37 patients, at least one actionable gene alteration was found, and 18 of them did not receive therapy according to the results. 

**Figure 3:**
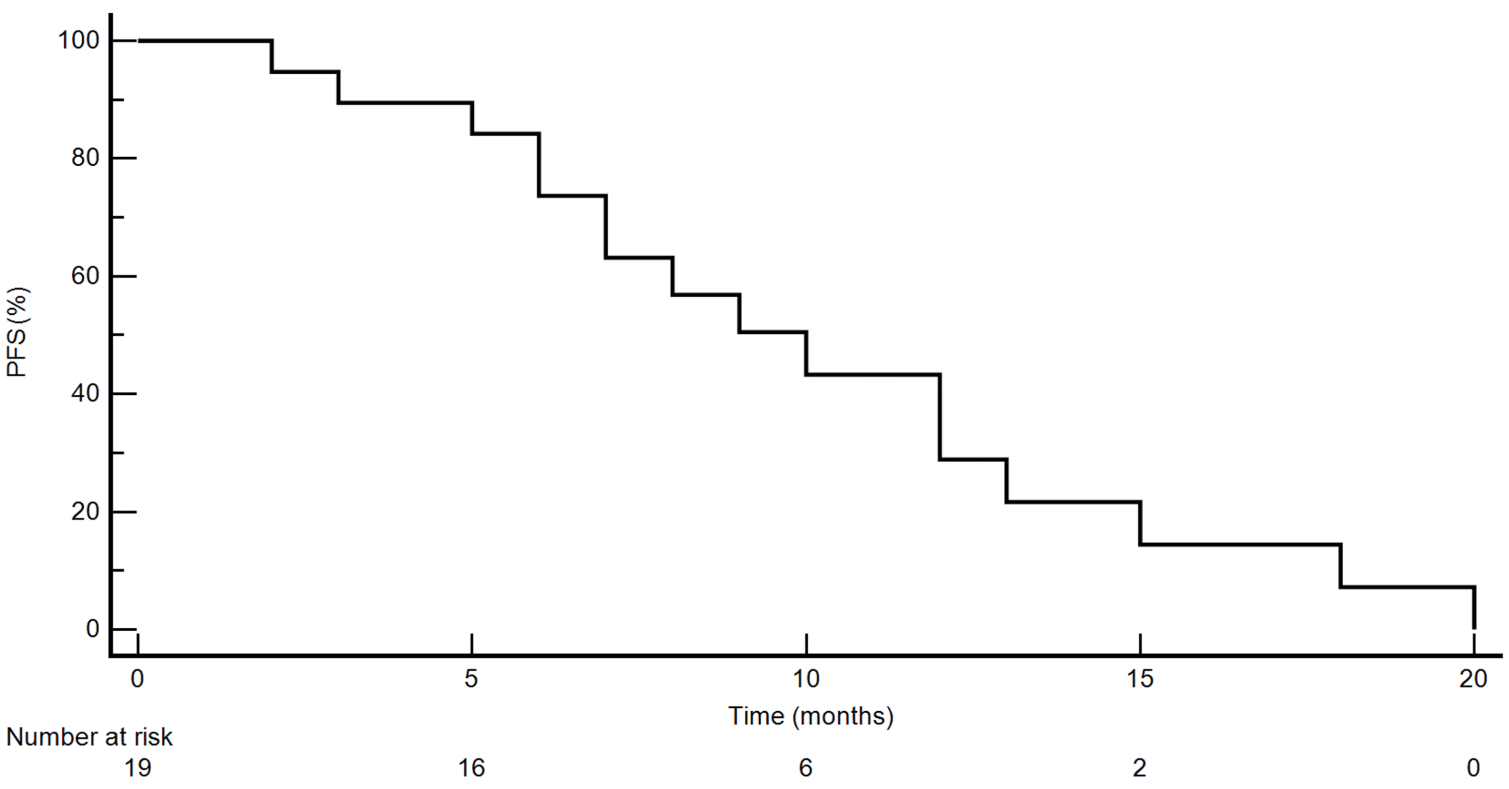
PFS rate for patients who received matched therapy PFS: progression-free survival

We performed a Kaplan-Meier analysis to determine the progression-free survival of patients who benefited from matched therapy after molecular profiling (Figure 3). The median PFS for patients who underwent matched targeted therapy was 10.1 months (with a 95% confidence interval of 6-13 months).

## Discussion

Out of all the tests conducted, 15 (20%) tests were unsuccessful due to two main reasons: (1) the patient's specimen did not provide an adequate amount of DNA and (2) because the patient's sample did not meet the minimum test criteria for tumor content. Specifically, for all samples (except liver), the requirement was that more than 20% of cells must originate from a malignant source. However, for liver samples, a minimum of 40% of the sample needed to contain malignant cells for the test to be considered valid. The success rate is similar to that of other studies, reporting a success rate over 75%-80% or higher [9-12]. In our opinion, adequate communication and coordination between the oncologist and the physician who provide the sample and also with the laboratory and the pathologist could help in coordinating the sampling procedure with the technical requirements of NGS testing, thereby minimising the likelihood of insufficient tissue or inadequate fixation for analysis.

There were 9% liquid biopsies and 91% tissue biopsies. The median number of previous lines of treatment was 2. Most samples were derived from metastatic sites, with only 29 samples obtained from the primary tumor. Among the biopsied metastatic lesions, liver (40%) and lung (11%) were the most frequent locations.

Out of all 66 patients tested, 55 (83%) had at least one genetic alteration, two had no genetic alterations found, and for nine, the test had failed. The most frequent genetic alteration was TP53 (53% of the successful tests), which is consistent with other studies that investigated this aspect [4,5], followed by KRAS (25% of the successful tests) and BRCA1/2 (20%) mutations, which is different from other studies that had PIK3CA as one of the most frequent mutations [5,9].

There were some cases where we enrolled patients with previously determined pathogenic mutations that progressed on targeted therapy, to determine the specific resistant mutations and the possibility of switching to another treatment. For example, a 71-year-old female diagnosed with metastatic NSCLC harbouring EGFR 19 deletion progressed on a first-generation tyrosine kinase inhibitor. After that we identified T790M mutation, and she was switched to osimertinib. She progressed after 27 months of therapy, so the tumor board decided to perform a NGS from a tissue sample of a new hepatic lesion. The tumor showed EGFR C797S, T790M, exon 19 deletion (T751_I759>N), CDK4 amplification, MDM2 amplification, CDKN2A/B loss, MTAP loss, low MSI, low TMB and negative PD-L1. Therefore, with no other effective targeted therapy found, the tumor board decided to switch the treatment to chemotherapy.

The median TMB of the successful tests was 7. In all the 66 patients enrolled in the cohort, there were only nine cases (14%) who had a high TMB: three patients had NSCLC, two had urothelial cancer, two were cutaneous melanoma cases, one patient had SCLC and one ovarian cancer. These results are comparable to another study that analysed the TMB distribution, where lung cancer was also the most frequent solid tumor that showed a high TMB [5]. Out of all, seven received treatment with an immune checkpoint inhibitor (ICI). One patient died soon after the test results and one of them received targeted therapy according to the genetic alterations found. It is to be mentioned that one of the patients was already under treatment with an ICI when the test was ordered. Only one patient presented with a high MSI, a 66-year-old male patient with metastatic melanoma, who also had a high TMB status and benefited from treatment with an ICI. In 11 out of 66 (17%) patients, PD-L1 expression could not be determined. For the others, 54% (36) had a negative score, 23% (15) had a low positive result, only 3% (two patients) had a moderate positive result and only 3% had a high positive result. Both patients with a high positive result were diagnosed with NSCLC.

There were 37 patients (56%) with actionable alterations found, from which 14 received matched therapy and four patients were enrolled in clinical trials. The other 18 did not receive matched therapy for different reasons. There were many cases in which the patients did not receive the matched therapy because of the rapid progression of their disease that led to a very poor performance status or even death. Others did not receive matched therapy because the drugs available were not reimbursed in our country. There are many factors that limited the number of patients who would benefit from matched therapy. Some of the aspects that can be taken into account include the limited access to targeted agents and to clinical trials, the limitations of the targeted agents available, the heterogeneity of the tumor that cannot be assessed by one tissue sample and also the financial issues [13].

From all the 66 patients tested, 23% received matched therapy. These results are similar with the ones observed in other studies or even higher than some other reports, where it was below 20% [4,5,9,10]. This percentage highlights one of the important problems related to NGS analysis, which is determining the optimal time to order the testing, maybe in earlier treatment of the metastatic disease, so that the patients can get the maximum benefit from it.

There were only four patients who were enrolled in a clinical trial based on the molecular testing results. This low number is most likely because of the scarcity of clinical trials in Eastern European countries and the low possibility to enroll the patients in clinical studies abroad due to personal/financial reasons.

The Kaplan-Meier analysis demonstrated that the median PFS for patients who underwent matched targeted therapy was 10.1 months (with a 95% confidence interval of 6-13 months). This result is a bit lower compared with other studies where the median PFS for the patients who benefited from treatment guided by genomic test results was around 12 months [4,5].

There were six patients (31.5%) who benefited from the targeted treatment for a duration exceeding one year: three of them showed a high TMB and received immunotherapy in later lines: two had urothelial cancer and one had NSCLC. Additionally, two patients with breast cancer, both carrying BRCA1 mutations (ESCAT IA), received olaparib in later lines. Furthermore, one patient diagnosed with vulvar Paget's disease presented with HER2 mutation and underwent HER2 targeted therapy.

These results emphasize the importance of more carefully selecting patients who require next-generation sequencing tests, and conducting these tests earlier in the course of disease progression so that the patients have a better chance of receiving a new line of treatment before the poor performance status constrains the therapeutic options.

Limitations

Despite being the sole source of available data on the experience with tumor profiling in cancer patients in Romania, this study has several limitations. This series represents a single-institution analysis and it encompasses only 66 patients, with diverse solid tumor types. The small number of patients and the diversity in the patient population mirrors the circumstances of other genomic profiling single-centre studies [4]. Additionally, this series constitutes a preliminary analysis of a single-institution experience aimed at gaining insights into the novel technology, its potential benefits, and its limitations. The results derived from a single-centre experience, while informative, inherently carry limitations in terms of generalizability due to the unique patient population and specific institutional practices. However, these findings can serve as a catalyst for encouraging and informing the development of a larger, multicentered analysis.

## Conclusions

The tests conducted exhibited a success rate of 80%, indicating that approximately one out of every five tests resulted in failure. Therefore, it has become imperative to explore alternative techniques for the selection and transportation of probes, with the aim of improving their efficiency and effectiveness.

The moderate success of personalized medicine using NGS testing highlights the importance of evaluating the factors that could lead to further improvement. There is also the argument about whether all the testing and expensive medications are cost-effective, and whether satisfactory clinical effectiveness can be derived from all the testing and administering expensive new drugs. Considering the complexity of a NGS report and the cases that needed thorough discussions and molecular biology experts, we underscore the importance of molecular tumor boards that can properly assess the results for each patient and explore the potential therapeutic opportunities with approved or new drugs under investigation.

From all the data that were derived from this analysis and from other publications, personalized medicine is something to look forward to but still something that requires refinement. It is clear that there is some benefit for a group of patients, and it is important to improve the selection of patients, the right timing for the testing, and the access to clinical trials. Also, further exploration is needed to determine the most efficient testing platform, and to improve the cost-effectiveness of personalized medicine especially in developing countries.
