# Correction: Role of Tumor Molecular Profiling With FoundationOne®CDx in Advanced Solid Tumors: A Single-Centre Experience From Romania

**DOI:** 10.7759/cureus.c211

**Published:** 2025-02-14

**Authors:** Ana Maria Popa, Mihaela Andreea Stejeroiu, Cristian Iaciu, Mihaela Olaru, Cristina Orlov Slavu, Andreea Parosanu, Ioana Miruna Stanciu, Cristina Pirlog, Simina Pavel, Cornelia Nitipir

**Affiliations:** 1 Medical Oncology, Elias Emergency University Hospital, Bucharest, ROU; 2 Oncology, Carol Davila University of Medicine and Pharmacy, Bucharest, ROU

This article has been corrected at the request of the submitting author to add an affiliation for Ana Maria Popa:

Oncology, Carol Davila University of Medicine and Pharmacy, Bucharest, Romania

